# Systematic engineering enables efficient biosynthesis of L-phenylalanine in *E. coli* from inexpensive aromatic precursors

**DOI:** 10.1186/s12934-023-02282-0

**Published:** 2024-01-05

**Authors:** Mengzhen Nie, Jingyu Wang, Zeyao Chen, Chenkai Cao, Kechun Zhang

**Affiliations:** 1 Zhejiang University, Hangzhou, 310027 Zhejiang China; 2 Center of Synthetic Biology and Integrated Bioengineering, School of Engineering, Westlake University, Hangzhou, 310030 Zhejiang China

**Keywords:** L-phenylalanine, Aromatic precursors, Benzyl alcohol, Benzaldehyde, Engineering, *Escherichia coli*

## Abstract

**Background:**

L-phenylalanine is an essential amino acid with various promising applications. The microbial pathway for L-phenylalanine synthesis from glucose in wild strains involves lengthy steps and stringent feedback regulation that limits the production yield. It is attractive to find other candidates, which could be used to establish a succinct and cost-effective pathway for L-phenylalanine production. Here, we developed an artificial bioconversion process to synthesize L-phenylalanine from inexpensive aromatic precursors (benzaldehyde or benzyl alcohol). In particular, this work opens the possibility of L-phenylalanine production from benzyl alcohol in a cofactor self-sufficient system without any addition of reductant.

**Results:**

The engineered L-phenylalanine biosynthesis pathway comprises two modules: in the first module, aromatic precursors and glycine were converted into phenylpyruvate, the key precursor for L-phenylalanine. The highly active enzyme combination was natural threonine aldolase LtaE_P.p_ and threonine dehydratase A8H_B.t_, which could produce phenylpyruvate in a titer of 4.3 g/L. Overexpression of gene *ridA* could further increase phenylpyruvate production by 16.3%, reaching up to 5 g/L. The second module catalyzed phenylpyruvate to L-phenylalanine, and the conversion rate of phenylpyruvate was up to 93% by co-expressing PheDH and FDH^V120S^. Then, the engineered *E. coli* containing these two modules could produce L-phenylalanine from benzaldehyde with a conversion rate of 69%. Finally, we expanded the aromatic precursors to produce L-phenylalanine from benzyl alcohol, and firstly constructed the cofactor self-sufficient biosynthetic pathway to synthesize L-phenylalanine without any additional reductant such as formate.

**Conclusion:**

Systematical bioconversion processes have been designed and constructed, which could provide a potential bio-based strategy for the production of high-value L-phenylalanine from low-cost starting materials aromatic precursors.

**Supplementary Information:**

The online version contains supplementary material available at 10.1186/s12934-023-02282-0.

## Background

L-phenylalanine is a valuable amino acid with multiple industrial applications [1]. It is extensively used in dietary supplements, feed, cosmetics, and chemical industries [2–4]. In addition, L-phenylalanine is widely used in the synthesis of pharmaceutically active compounds, such as cephalosporin antibiotics, anticancer metallodrugs, and HIV protease inhibitors [5–7], resulting in a worldwide steadily increasing demand for L-phenylalanine.

**Fig. 1:**
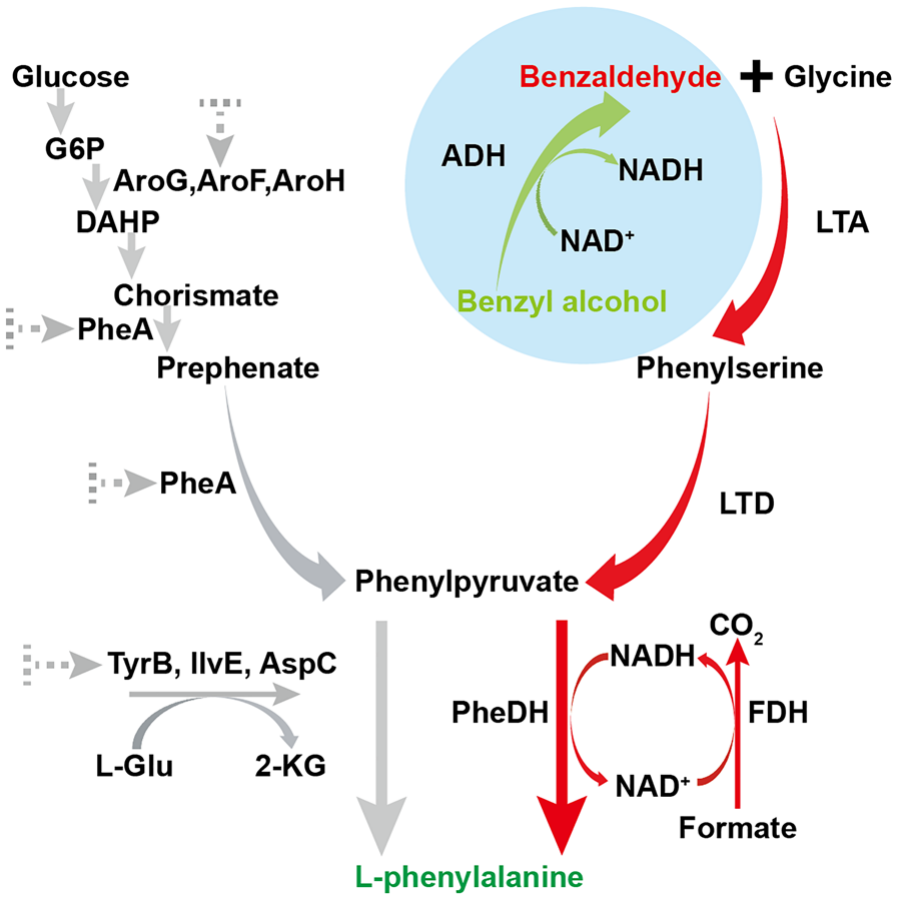
Design the artificial biosynthetic pathways for L-phenylalanine production from aromatic precursors (benzaldehyde or benzyl alcohol) and glycine. Gray arrows indicated the natural biosynthesis pathway of L-phenylalanine in *E. coli.* The green and red arrows demonstrated the novel pathway. ADH alcohol dehydrogenase, LTA threonine aldolase, LTD threonine dehydratase, PheDH phenylalanine dehydrogenase, FDH formate dehydrogenase. The dotted gray arrows  indicate the repression and inhibition of the relevant genes in native pathway. Metabolites abbreviations: G6P glucose 6-phosphate, DAHP 3-deoxy-d-arabino- heptulosonate-7-phosphate, L-Glu L-glutamate, 2-KG 2-ketoglutarate

Given the widespread applications and growing demands of L-phenylalanine, various strategies have been proposed for the production of L-phenylalanine. L-phenylalanine is primarily produced through chemical, microbial, or enzymatic processes [8]. Traditional chemical synthesis methods rely on expensive transition metals as catalysts and result in the accumulation of toxic by-products [9]. The microbial processes generate less environmental pollution than chemical synthesis [10–12]. Production of L-phenylalanine from glucose with relatively high titers in *E. coli* has been achieved in previous reports [7, 13–15]. However, the typical biosynthesis pathway in *E. coli* (Fig. 1 left) involves lengthy reaction steps (more than 15 steps) and tightly complex feedback regulation, which limits the practical yield of L-phenylalanine production [16–19]. Therefore, there is a need to find alternative substrates and establish a succinct, cost-effective pathway for L-phenylalanine production.

The aromatic chemicals (benzaldehyde and benzyl alcohol) are attractive candidates for L-phenylalanine production. These two chemicals are inexpensive with a price of ~$2/kg (benzyl alcohol) and ~$2.5/kg (benzaldehyde), respectively [20, 21]. The aromatic ring could provide the bulky carbon backbone for L-phenylalanine [22, 23]. In addition, the theoretical yield of aromatic precursor to L-phenylalanine is 100 mol%. Some researchers have reported that using threonine aldolases (TAs) and threonine deaminases (TDs) combination could catalyze the conversion of aldehydes and small amino acids into keto acids [24–26]. The conversion of phenylpyruvate to L-phenylalanine by the L-amino acid dehydrogenase or aminotransferase system has been investigated by previous works [27, 28]. One report demonstrated that benzaldehyde could be used to synthesize L-phenylalanine by multi-enzyme-coupled reactions [24]. However, the production of L-phenylalanine with benzyl alcohol as the precursor has not been published to date.

Compared with benzaldehyde, benzyl alcohol is less toxic to cells and has less effect on enzyme activity and cell growth, making the process more feasible [29]. In addition, Benzyl alcohol is more stable than benzaldehyde, which is readily oxidized to benzoic acid on exposure to air at room temperature [30]. Moreover, benzyl alcohol has better solubility than benzaldehyde, which can avoid the use of cosolvent such as DMSO, and reduce production costs. Benzyl alcohol dehydrogenase from *Pseudomonas putida* (XylB_P.p_) has been reported to be highly active in converting benzyl alcohol to benzaldehyde, while also providing cofactor NADH for L-phenylalanine production by reducing NAD^+^ [31].

Here, the work aimed to develop the biosynthetic processes of L-phenylalanine from inexpensive and readily available aromatic precursors (as shown in Fig. 1 right). We first enable the key intermediate phenylpyruvate production by screening and co-expressing the enzymes threonine aldolase LtaE_P.p_ and L-phenylalanine dehydratase A8H_B.t_. Introducing the enamine deaminase RidA could further improve the titer of phenylpyruvate by 16.3%, reaching up to 5 g/L. The conversion rate of phenylpyruvate to L-phenylalanine was 93% by a recombinant redox cycle including phenylalanine dehydrogenase (PheDH) and formate dehydrogenase (FDH^V120S^). Synthesis of L-phenylalanine from benzaldehyde was performed, resulting in an L-phenylalanine titer of 1.7 g/L and a benzaldehyde conversion rate of 69%. Moreover, we constructed a cofactor self-sufficient pathway for L-phenylalanine production from benzyl alcohol, a process that NADH/NAD^+^ in different redox states are interconverted via the enzymes pair XylB_P.p_ and PheDH without another regenerating enzyme, and does not require the reductant formate, which results in a cleaner bioconversion system. In summary, this work describes a succinct and feasible biosynthesis of L-phenylalanine from inexpensive aromatic precursors (benzyl alcohol or benzaldehyde).

## Results and discussion

### Identification of enzymes to produce the key intermediate phenylpyruvate

**Fig. 2:**
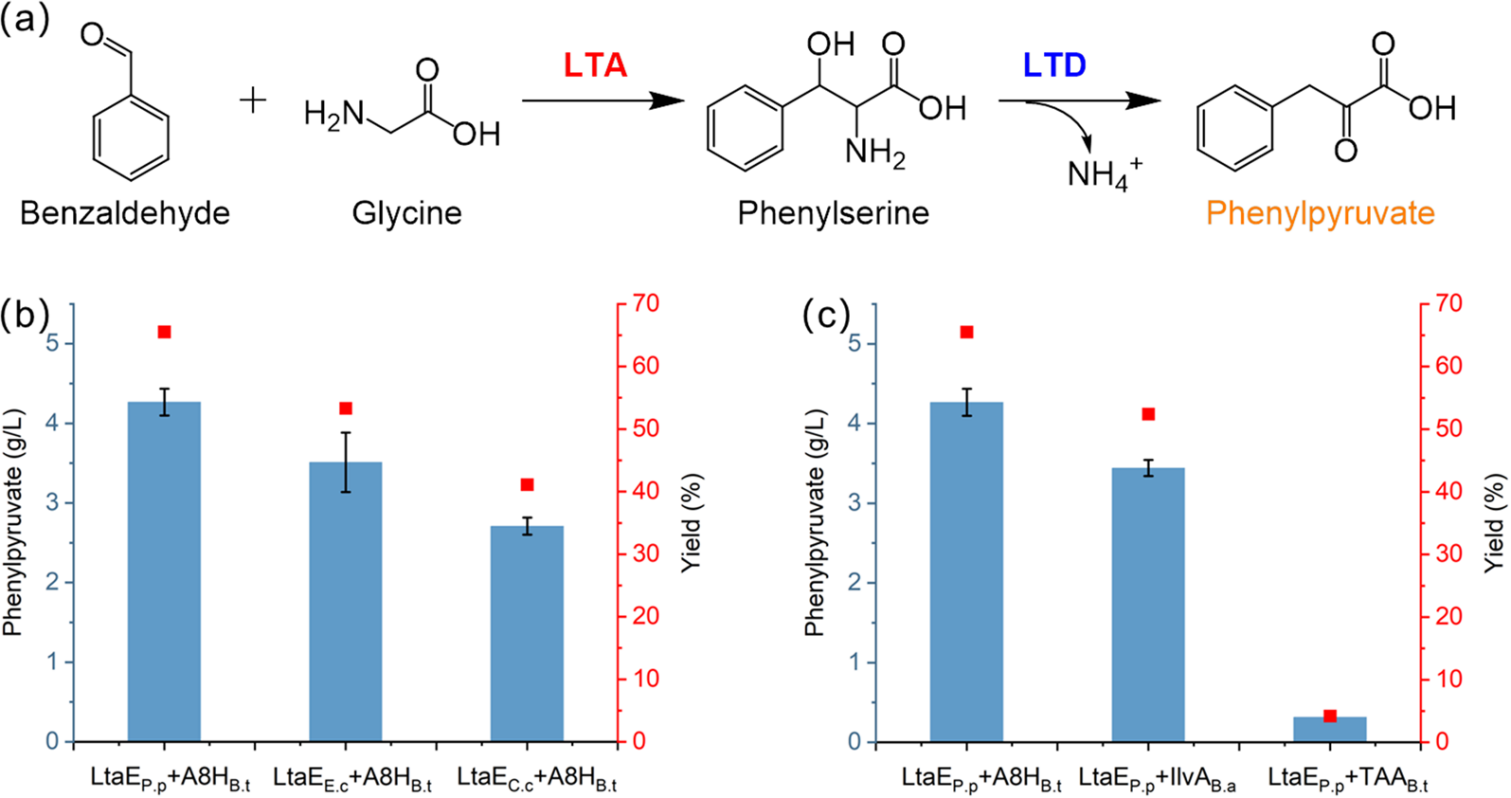
Phenylpyruvate production using different combinations of threonine aldolase (LTA) and threonine dehydratase (LTD). **a** Phenylpyruvate production pathway from benzaldehyde and glycine catalyzed by LTA and LTD. **b** The effect of different threonine aldolases (LtaE_P.p_, LtaE_E.c_, LtaE_C.c_) on phenylpyruvate production. **c** The effect of different L-phenylalanine dehydratase (A8H_B.t_, IlvA_B.a_, TAA_B.t_) on phenylpyruvate production. Error bars are the standard deviation for three independent experiments

The validation of this L-phenylalanine pathway started with building a well-behaved chassis for phenylpyruvate production, which is the key precursor for L-phenylalanine production. We used benzaldehyde and glycine as substrates to screen natural high-active enzymes threonine aldolase (LTA) and threonine dehydratase (LTD) (Fig. 2a). To convert benzaldehyde and glycine to phenylserine, we screened three LTAs from *Pseudomonas putida* (LtaE_P.p_), *E. coli* (LtaE_E.c_), and *Caulobacter crescentus* CB15 (LtaE_C.c_), and cloned them into plasmids pPLA-1, pPLA-2, and pPLA-3, respectively (Table 1). These plasmids also carried a threonine/serine dehydratase from *Burkholderia thailandensis* (A8H_B.t_), for screening based on phenylpyruvate production, and were transformed into wild-type BW25113 individually, to obtain strains M1, M2, and M3 (Table 1). We used 4.2 g/L (40mM) benzaldehyde and 20 g/L glycine as cosubstrate, as shown in Fig. 2b, the strains expressing LtaE_P.p_, LtaE_E.c_, and LtaE_C.c_ produced phenylpyruvate at a titer of 4.3 g/L (26 mM), 3.5 g/L (21 mM), and 2.7 g/L (16.4 mM) within 24 h, respectively (column #1, #2, #3 in Fig. 2b). Notably, all of these enzymes are promiscuous enough to catalyze the bioconversion of benzaldehyde and glycine into phenylpyruvate. These results indicated that LtaE_P.p_, among the three investigated enzymes, was the best natural LTA for benzaldehyde conversion.

**Table 1:** Strains and plasmids used in this study

Strains/plasmids	Phenotype	Source
Strains		
BW25113	*Δ(araD-araB)567ΔlacZ4787(::rrnB-3) ΔlacZ4787(::rrnB-3) Δ(rhaD-rhaB)568 hsdR514*	CGSC
*E. coli* DH5α	Host for plasmid construction	This study
M1	BW25113 [pPLA-1]	This study
M2	BW25113 [pPLA-2]	This study
M3	BW25113 [pPLA-3]	This study
M4	BW25113 [pPLA-4]	This study
M5	BW25113 [pPLA-5]	This study
M6	BW25113 [pPLA-6]	This study
M7	BW25113 [pPLA-7]	This study
M8	BW25113 [pPLA-6, pPLA-7]	This study
M9	BW25113 [pPLA-6, pPLA-8]	This study
Plasmids		This study
pPLA-1^a^	pZE-*P*_*LlacO1*_*-ltaE*_*P.p*_*-A8H*_*B.t*_	This study
pPLA-2^a^	pZE-*P*_*LlacO1*_*-ltaE*_*E.c*_*-A8H*_*B.t*_	This study
pPLA-3^a^	pZE-*P*_*LlacO1*_*-ltaE*_*C.c*_*-A8H*_*B.t*_	This study
pPLA-4^a^	pZE-*P*_*LlacO1*_*-ltaE*_*P.p*_*-ilvA*_*B.a*_	This study
pPLA-5^a^	pZE-*P*_*LlacO1*_*-ltaE*_*P.p*_*-TAA*_*B.t*_	This study
pPLA-6^a^	pZE-*P*_*LlacO1*_*-ltaE*_*P.p*_*-A8H*_*B.t*_-*ridA*	This study
pPLA-7^a^	pZA-*P*_*LlacO1*_*-fdh-pdh*	This study
pPLA-8^a^	pZA-*P*_*LlacO1*_*-xylB*_*P.p*_*-pdh*	This study

^a^The isopropyl-β-D-thio-galactoside (IPTG) was required to induce the overexpression of introduced genes in plasmids

In addition to LTAs, we also investigated other LTDs for α,β-elimination to see which combination would produce the maximal titer of phenylpyruvate. We cloned genes coding L-threonine dehydratase from *Burkholderia ambifaria* (*ilvA*_*B.a*_) and ammonia-lyase from *Burkholderia thailandensis* (*TAA*_*B.t*_) individually after the gene encoding LtaE_P.p_ to build an expression cassette on a high-copy plasmid, named pPLA-4, and pPLA-5, respectively. The strains transformed with pPLA-4 and pPLA-5 (Table 1, strains M4 and M5) could produce 3.4 g/L (21 mM) and 0.3 g/L (1.8 mM) phenylpyruvate within 24 h, respectively (column #2, #3 in Fig. 2c). Overall, the highly active natural enzyme combination was LtaE_P.p_ and A8H_B.t_ for the phenylpyruvate production from benzaldehyde and glycine, resulting in a benzaldehyde conversion rate of 65%. In a previous report, the conversion of benzaldehyde and glycine to phenylpyruvate was improved from 23% (wide-type threonine deaminase) to 88% (mutated by rational protein engineering) [24]. In this respect, further directed evolution in A8H_B.t_ could significantly enhance the production of phenylpyruvate in our future work.

### Effect of temperature and pH on phenylpyruvate production

**Fig. 3:**
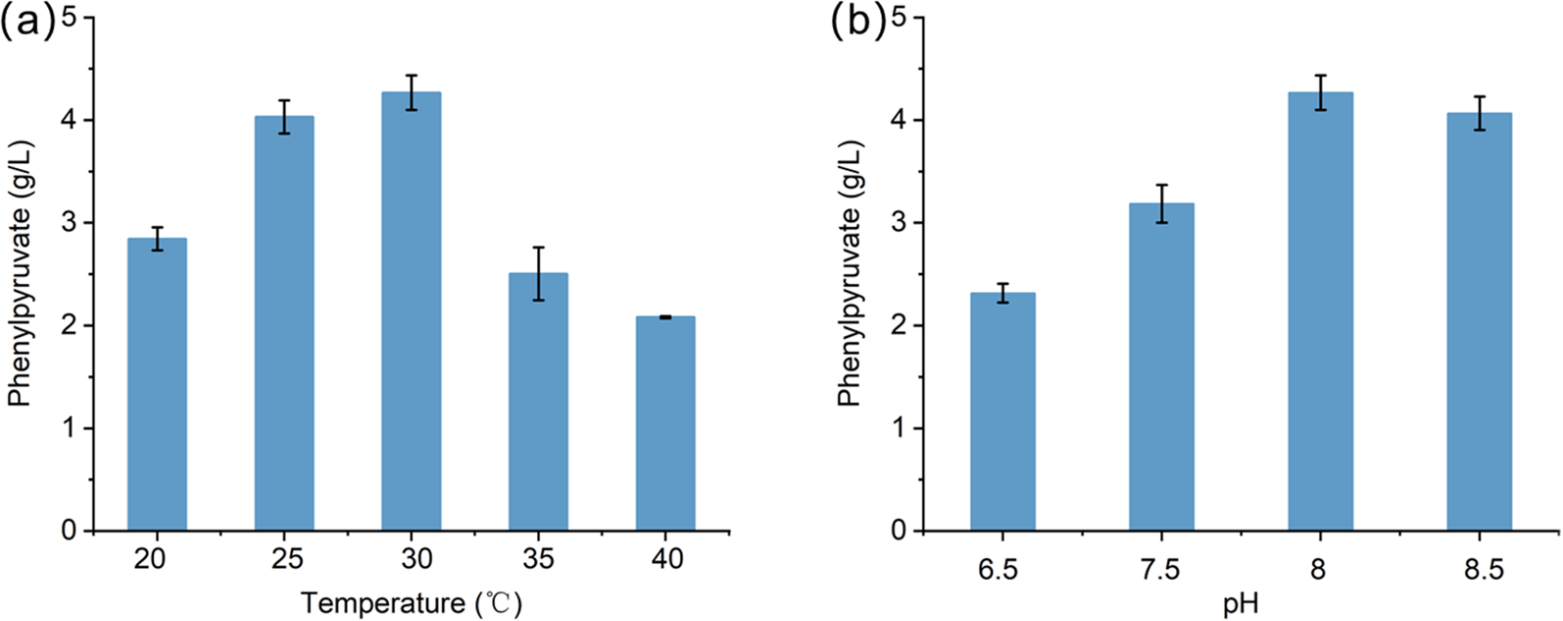
Effect of reaction conditions on the phenylpyruvate production by the enzyme cascade of LtaE_P.p_ and A8H_B.t_ combination. **a** The effects of reaction temperatures on the phenylpyruvate production. **b** The effects of reaction pH on the phenylpyruvate production. The following buffer systems were used: 100 mM Tris-HCl for pH 6.5, 7.5, 8.0, and 8.5. Error bars are the standard deviation for three independent experiments

The reaction conditions of the enzyme cascade catalytic system are important for production performance. To achieve a good conversion of reactants, the temperature and pH of the reaction system need to be optimized [32]. Preliminary analysis of the plasmid pPLA-1 showed the highest activity toward phenylpyruvate production, and we used strain M1 to analyze the effects of different reaction conditions on phenylpyruvate production. As shown in Fig. 3a, the activity of this enzyme cascade was optimum at 30 ℃. When the assay temperature was set at 20 ℃, 25 ℃, 35 ℃ and 40 ℃, the phenylpyruvate titer was reduced to 2.84 g/L (17 mM), 4 g/L (24.1 mM), 2.5 g/L (15.2 mM) and 2.1 g/L (12.8 mM), respectively, about 66%, 93%, 58% and 48% of that value at 30 ℃. Under different pHs in the reaction mixture (Fig. 3b), enzyme activity of LtaE_P.p_ and A8H_B.t_ combination was optimum at pH 8.0, with 4.3 g/L (26 mM) phenylpyruvate detected. The enzyme activities were almost not affected when the pH value was up to 8.5, 4.0 g/L (24.3 mM) phenylpyruvate was produced. The titer of phenylpyruvate decreased to 3.2 g/L (19.4 mM) at pH 7.5, and 2.3 g/L (14 mM) at pH 6.5, respectively. These results indicated that the enzyme activities of LtaE_P.p_ and A8H_B.t_ were highly dependent on temperature and pH. Further experiments were conducted at the optimum conditions of 30 ℃ and pH 8.0.

## Overexpression of RidA could further increase phenylpyruvate production

Threonine dehydratase, as a Pyridoxal 5’-phosphate (PLP) dependent enzyme, will generate active imine/enamine intermediates that are converted into keto acid by a protein of the members of the RidA family, which were recently shown to be enamine deaminases [33–35]. Lacking RidA would decrease the activity of the PLP-dependent transaminase enzyme IlvE in *S. enterica* strains [36]. It has been reported that the presence of RidA could increase the rate of 2-ketobutyrate formation from threonine by the enzyme IlvA [37]. However, there was no previous report about the effect of gene *ridA* on phenylpyruvate production catalyzed by threonine dehydratase.

**Fig. 4:**
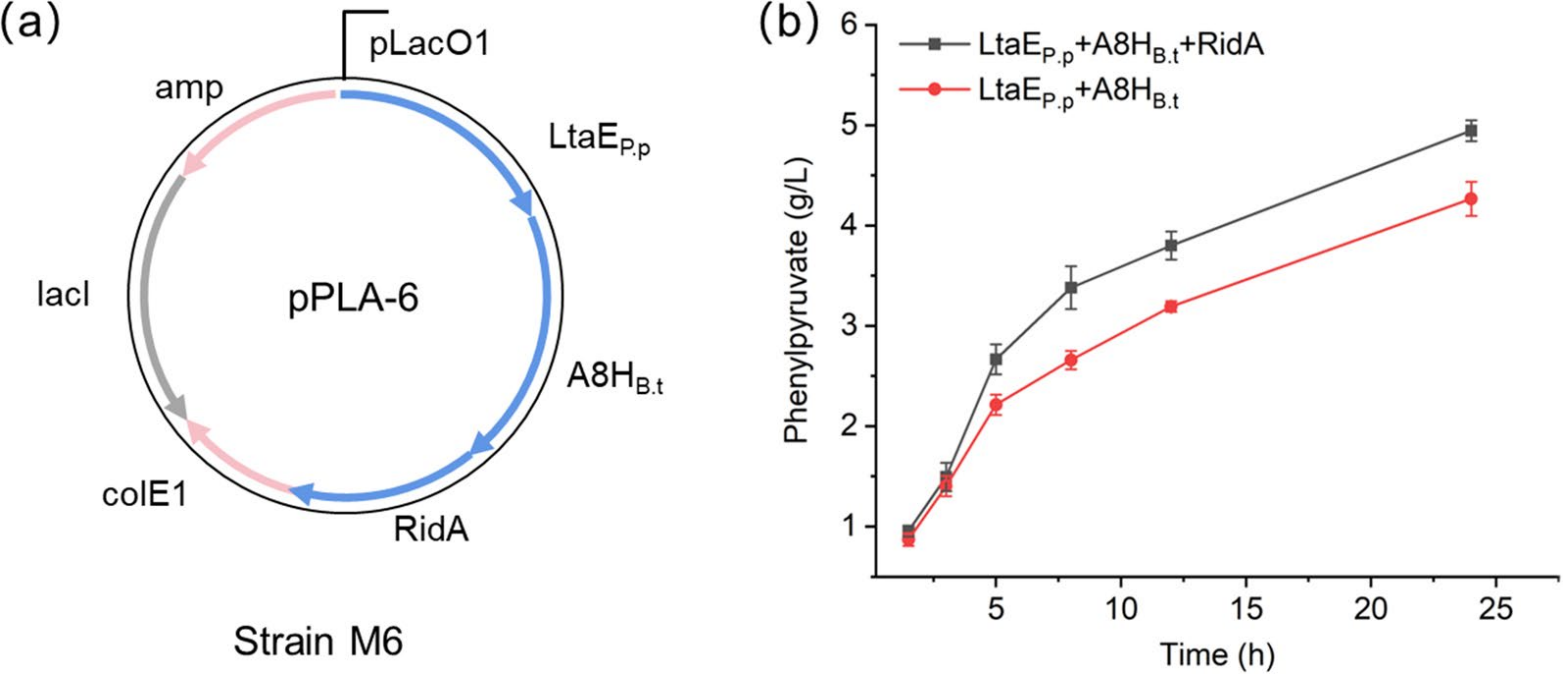
The construction and evaluation of RidA strain M6. **a** Construction of strain M6 with plasmid pPLA-6 overexpressing RidA. **b** Effect of enzyme RidA on the conversion of benzaldehyde to phenylpyruvate. Error bars are the standard deviation for three independent experiments

To address this issue, we cloned gene *ridA* after the gene encoding LtaE_P.p_ and A8H_B.t_ to build the plasmid pPLA-6 (Fig. 4a), and transformed it into BW25113, yielding strain M6. The fermentation results showed that after overexpression of gene *ridA*, the titer of phenylpyruvate was increased to 5.0 g/L (30.1 mM) within 24 h (Fig. 4b), and the practical conversion rate of benzaldehyde was up to 77%. These results confirmed that overexpression of gene *ridA* could provide more enamine deaminase to enhance phenylpyruvate formation. However, even with the optimized cascade system, it still cannot lead to a complete conversion of benzaldehyde, which is possibly due to the relatively low activity of dehydratase A8H_B.t_ for the second reaction. For enzyme cascade processes, the second step with high activity can pull the substrates flux into the desired synthesis pathway, improving the overall conversion of the reaction system [38]. We can further improve the dehydratase activity through directed evolution in the future [24, 39]. Besides, we can remove the target genes in the host to minimize the effect of competing endogenous pathways and improve the introduced synthetic pathway performance [40, 41].

### Production of L-phenylalanine from phenylpyruvate

**Fig. 5:**
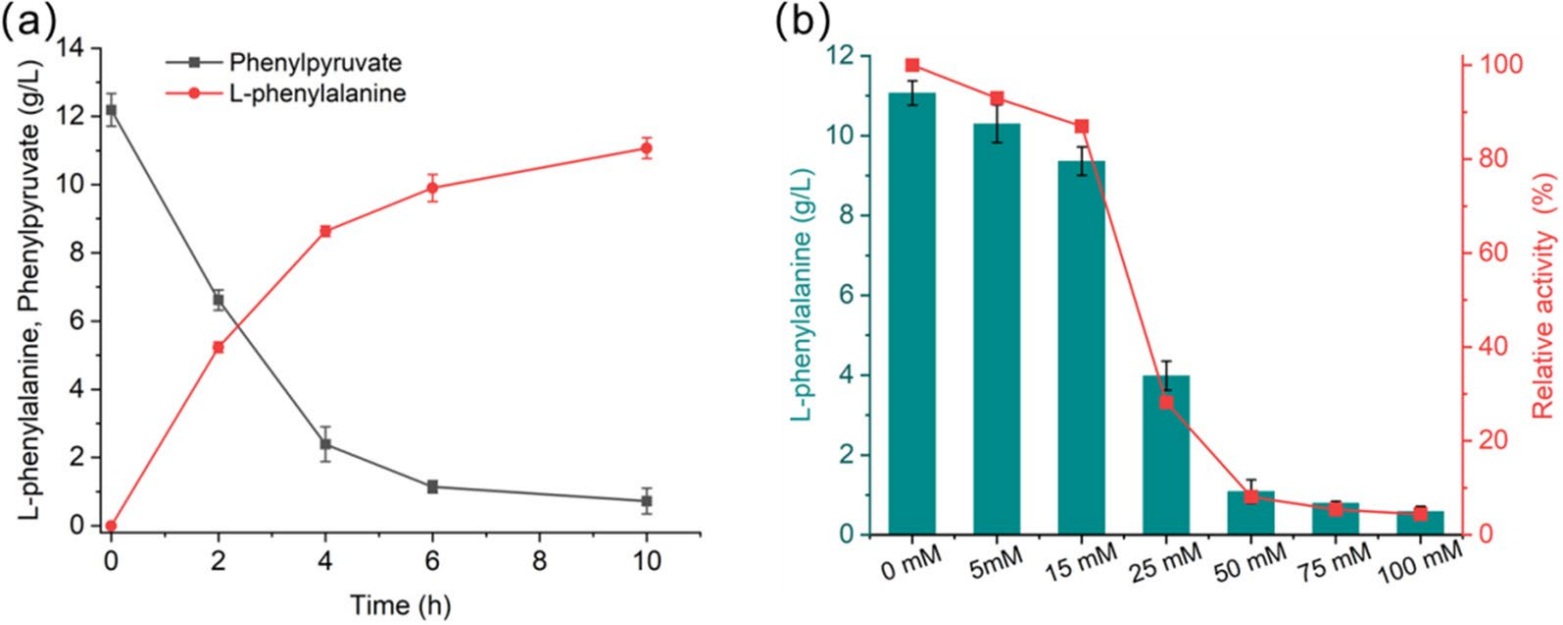
Biotransformation of phenylpyruvate into L-phenylalanine. **a** L-phenylalanine production from phenylpyruvate by coexpressing *Bacillus badius* phenylalanine dehydrogenase (PheDH) and *Candida boidinii* formate dehydrogenase (FDH^V120S^). FDH was used for cofactor NADH regeneration. **b** The tolerance of the key enzymes PheDH and FDH^V120S^ to benzaldehyde. Error bars are the standard deviation for three independent experiments

Phenylalanine dehydrogenase from *Bacillus badius* (PheDH, encoded by gene *pdh*) was selected to promote the conversion of phenylpyruvate into L-phenylalanine [42]. Enzymatic dehydrogenation is usually performed in the presence of stoichiometric amounts of coenzyme (NADH or NADPH), implying the coenzyme cofactor must be recycled during the conversion. *Candida boidinii* formate dehydrogenase (FDH) has been employed as a workhorse for efficient NADH regeneration for decades, which can be used in a broad pH range of 6 ~ 9 [43–45]. In a previous report, the mutant V120S has been shown to greatly improve the activity and stability of enzyme FDH in the catalytic reaction [46]. To ensure sufficient cofactor NADH supply, a recombinant plasmid pPLA-7 expressing enzymes PheDH and FDH^V120S^ was constructed and transformed into BW25113, yielding strain M7. The conversion performance of *E. coli* with pPLA-7 (strain M7) was investigated with 0.07 M phenylpyruvate, 0.35 M formate, and 0.14 M NH_4_Cl at 30 ℃ (Fig. 5a). 11 g/L (0.066 M) L-phenylalanine was produced within 10 h, amounting to a conversion rate of 93% of phenylpyruvate. The results showed that the enzyme cascade of PheDH and FDH^V120S^ display high efficacy in the conversion of phenylpyruvate to L-phenylalanine.

Then, the tolerance of the key enzymes PheDH and FDH^V120S^ to benzaldehyde was tested. We performed the same conversion of phenylpyruvate to L-phenylalanine with the addition of varying concentrations of benzaldehyde, as shown in Fig. 5b. As the benzaldehyde concentration increased to 5 mM and 15 mM, the relative activity of enzyme cascade PheDH and FDH toward phenylpyruvate could keep at 93% and 87%, respectively. When benzaldehyde concentration increased up to 25 mM, the L-phenylalanine titer was remarkably decreased by 67% compared with the control experiments (column #1 in Fig. 5b). These results indicated that the efficiency of enzyme cascade PheDH-FDH^V120S^ would be negatively affected by high concentrations of benzaldehyde. Therefore, concentration-limited feeding of benzaldehyde could be adopted to maintain high-yield conversion. In addition, detoxification can be achieved through evolution to improve the tolerance of enzymes to the substrate benzaldehyde [47].

### Biosynthesis of L-phenylalanine from aromatic precursor benzaldehyde

**Fig. 6:**
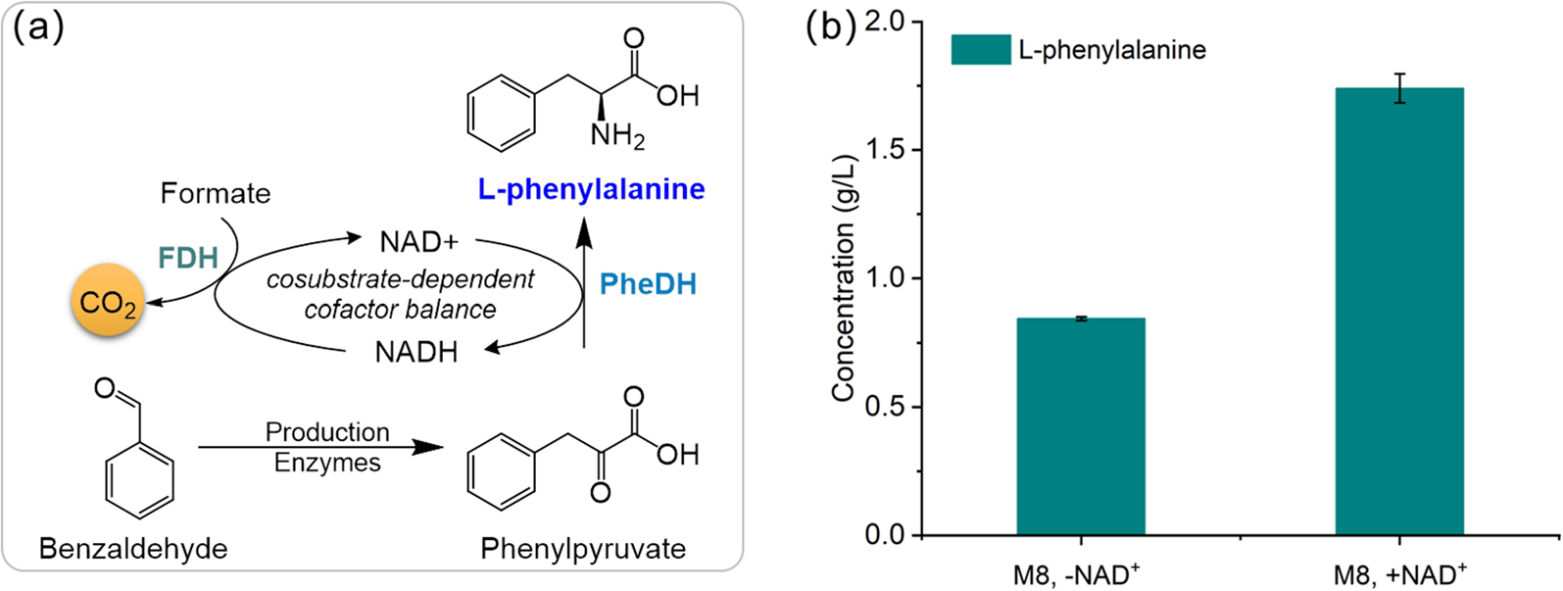
Biosynthesis of L-phenylalanine from aromatic precursor benzaldehyde. **a** Scheme showing the one-pot L-phenylalanine production from benzaldehyde with co-substrate formate for cofactor NADH regeneration. Production enzymes indicated the enzymes LtaE_P.p_, A8H_B.t_, and RidA that converted benzaldehyde to phenylpyruvate. **b** Biotransformation of the L-phenylalanine production in strain M8. Strain M8: BW25113 transformed with plasmids pPLA-6 and pPLA-7 to overexpress genes *ltaE*_*P.p*_, *A8H*_*B.t*_, *ridA, pdh, and fdh*. Error bars are the standard deviation for three independent experiments

Synthesis of L-phenylalanine from benzaldehyde and glycine was performed by using these two modules LtaE_P.p_-A8H_B.t_-RidA and PheDH- FDH^V120S^ in one host cell (as shown in Fig. 6a). Plasmids pPLA-6 and pPLA-7 were co-transformed into *E. coli* BW25113 to obtain strain M8. The L-phenylalanine production performance of strain M8 (Table 1) was investigated with 15 mM benzaldehyde, 150 mM glycine, and 150 mM formate at 30 ℃. As shown in Fig. 6b, without cofactor NAD^+^ addition, 0.84 g/L (5 mM) L-phenylalanine was detected after 24 h (column #1in Fig. 6b). The conversion rate of benzaldehyde was relatively low, only 34%. Employing the multi-enzyme cascade, the formation of the NAD^+^/NADH cofactor equilibrium was not as efficient as in the two-enzyme system [48]. To prove this hypothesis, we added 0.5 mM NAD^+^ under the same conversion condition, as expected, the titer of L-phenylalanine was significantly increased to 1.7 g/L (10 mM) after 24 h with a conversion of 69% (more than 2-fold increase, column #2 in Fig. 6b), implying that the addition of feasible amounts of cofactor could increase the yield and productivity for L-phenylalanine.

In a previous report [24], the titer of L-phenylalanine production from benzaldehyde via an enzyme cascade was up to 1.5 g/L by semisaturated mutation of CgTD for α,β-elimination and reaction conditions optimization. Therefore, we also tested the engineered threonine deaminase CgTD^F114A,R229T^ for L-phenylalanine production under our experimental conditions (Additional file 1: Table S1). It can be seen that the activity of wild-type A8H_B.t_ can be comparable to that of the CgTD^F114A,R229T^ for L-phenylalanine production. Therefore, L-phenylalanine production can be further improved in the future by the wild-type protein A8H_B.t_ adaptive evolution.

### Cofactor self-sufficient system for L-phenylalanine production from precursor benzyl alcohol

**Fig. 7:**
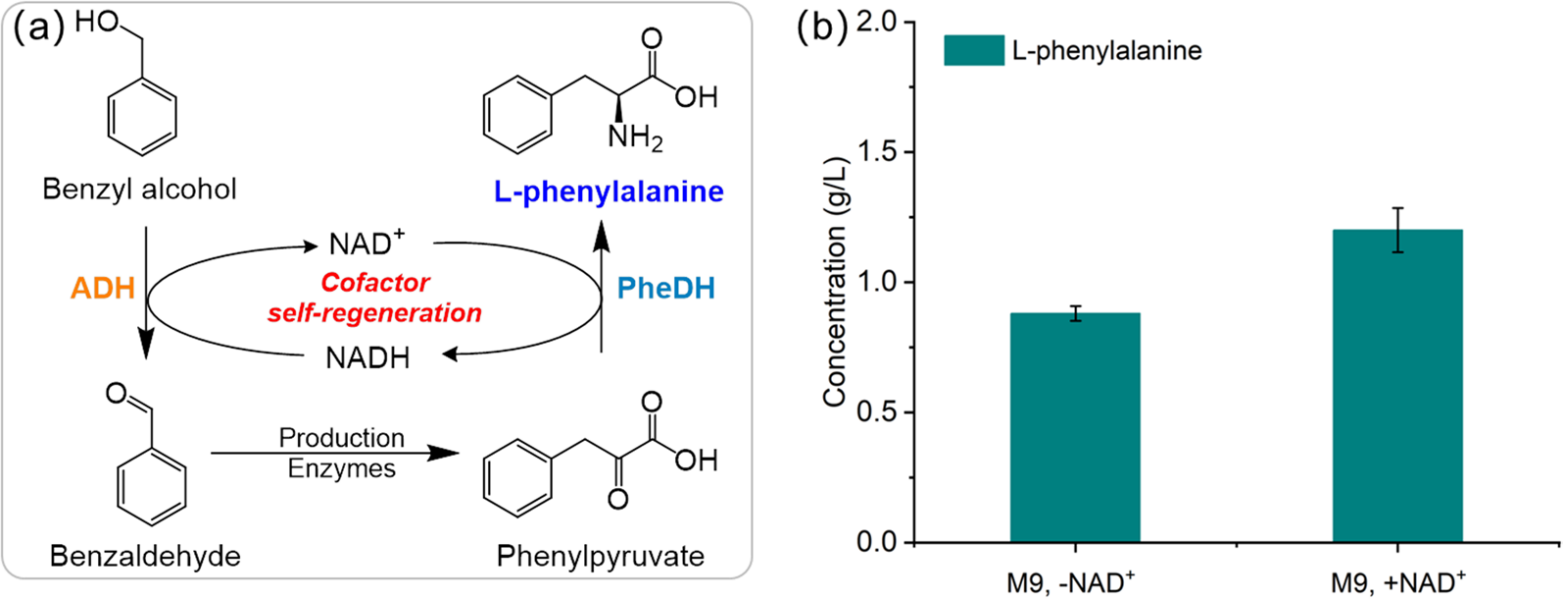
Biosynthesis of L-phenylalanine from aromatic precursor benzyl alcohol. **a** Scheme showing the one-pot L-phenylalanine production from benzyl alcohol with an engineering cofactor NADH self-regeneration system. Production enzymes indicated the LtaE_P.p_, A8H_B.t_, and RidA that converted benzaldehyde to phenylpyruvate. **b** Biotransformation of the L-phenylalanine production in strain M9. Strain M9: BW25113 transformed with plasmids pPLA-6 and pPLA-8 to overexpress genes *ltaE*_*P.p*_, *A8H*_*B.t*_, *ridA, pdh, and xylB*_*P.p*_. Error bars are the standard deviation for three independent experiments

As can be seen from Fig. 6a, the enzyme FDH^V120S^ was exploited for NADH balance by enzymatically oxidizing the sacrificial substrate formate into CO_2_, which not only complicates the L-phenylalanine production system but also increases the production cost. Therefore, based on these benzaldehyde conversion processes, we expanded the aromatic precursors to produce L-phenylalanine from another inexpensive aromatic compound benzyl alcohol. Benzyl alcohol can be oxidized into benzaldehyde by aryl-alcohol dehydrogenase, while obtaining NADH equivalents for reducing phenylpyruvate into L-phenylalanine. In addition, Benzyl alcohol is less toxic to enzyme activity and cell growth than benzaldehyde, making the process more feasible. Therefore, we tried to develop a bioconversion process for synthesizing L-phenylalanine from benzyl alcohol by rational design, which does not require additional cosubstrate as a reductant or another cofactor regenerating enzyme, achieving an important self-sufficient cofactor regeneration system (as shown in Fig. 7a).

Benzyl alcohol dehydrogenase from Pseudomonas putida (XylB_P.p_) has been reported to have a high activity toward benzyl alcohol [31]. We cloned the genes *xylB*_*P.p*_ and *pdh* into the plasmid pPLA-8. BW 25113 transformed with plasmids pPLA-6 and pPLA-8 (strain M9) was used for the conversion of benzyl alcohol and glycine into L-phenylalanine. With 15 mM benzyl alcohol and 150 mM glycine as co-substrates, this new enzyme cascade can accumulate 0.82 g/L (4.8 mM) and 1.1 g/L (6.7 mM) L-phenylalanine without or with 0.5 mM NAD^+^ addition, respectively (column #1, #2 in Fig. 7b). These results for the first time demonstrated a cofactor self-sufficient system to produce L-phenylalanine from aromatic precursor without any addition of reductant like formate. In the future, we can further optimize the bioconversion pathway by screening and evolving target enzymes with higher activity to further increase the efficiency of L-phenylalanine production from benzyl alcohol. In addition, metabolic engineering of the chassis cell could also be adopted to further improve the performance of L-phenylalanine production, such as increasing the uptake of substrate, and reducing substrate and product degradation [50].

## Conclusions

In this work, we developed and constructed an artificial biosynthesis pathway for L-phenylalanine production from aromatic precursors. To establish the pathway to phenylpyruvate, we first screened and expressed a high-activity natural enzyme combination including LtaE_P.p_ and A8H_B.t_, 4.3 g/L of phenylpyruvate was produced. Then, we confirmed that further overexpression of enzyme RidA could increase phenylpyruvate production by 16.3%, up to 5 g/L. The conversion of phenylpyruvate into L-phenylalanine was up to 93% by co-expressing PheDH and FDH^V120S^. Furthermore, the engineered *E. coli* overexpressing LtaE_P.p_, A8H_B.t_, RidA, PheDH, and FDH^V120S^ could produce 1.7 g/L L-phenylalanine from benzaldehyde, and the conversion rate of benzaldehyde was 69%. Finally, we constructed a new pathway for L-phenylalanine production from benzyl alcohol in a cofactor self-sufficient system. Based on overexpression LtaE_P.p_, A8H_B.t_, RidA, PheDH, and XylB_P.p_, 1.1 g/L L-phenylalanine was produced without the addition of reductant formate. The yield and productivity of the L-phenylalanine bioconversion system developed in this study still need to be improved in the future. Further work will focus on systematic protein screening or directed evolution to increase the activities of these key enzymes. Overall, we demonstrated a promising approach for the biosynthesis of L-phenylalanine from low-cost aromatic precursors.

## Materials and methods

### Strains and media

The details for all strains used in this study are shown in Table 1. The *E. coli* DH5α was used as the cloning host for plasmid construction. Unless otherwise specified, all strains were cultivated at 37 ℃ in LB media (10 g/L tryptone, 5 g/L yeast extract, and 10 g/L sodium chloride) medium with appropriate antibiotics.

### Biotransformation procedures

Three colonies of recombinant *E. coli* strain were cultivated for 12 h at 37 ℃ in LB medium with appropriate antibiotics. The culture was then inoculated (1% vol/vol) into a 250 mL flask with 50 ml fresh LB culture containing appropriate antibiotics. When the OD_600_ of the culture broth reached 0.6 ~ 0.8, 1 mM isopropyl β-D-1-thiogalactopyranoside (IPTG) was added to induce gene expression. The cells were inducted at 16 °C for 20 h and collected at 4 ℃ by centrifugation (7500×g, 5 min). Then, the strains were resuspended in the appropriate buffer to the desired density as resting cells for biotransformation.

Biotransformation of benzaldehyde and glycine to phenylpyruvate was conducted with resting cells of *E. coli* (M1, M2, M3, M4, M5, M6, the OD_600nm_ of the condensed cell was 50) in 2 ml Tris buffer (100 mM, pH 8.0), 50 µM PLP, at 220 rpm and 30 °C for 24 h. Biotransformation of phenylpyruvate to L-phenylalanine was conducted with resting cells of *E. coli* M7 (the OD_600nm_ of the condensed cell was 50) in 2 ml Tris buffer (100 mM, pH 8.0), 0.07 M phenylpyruvate, 0.35 M formate, and 0.14 M NH_4_Cl at 220 rpm and 30 °C for 24 h. Biotransformation of benzaldehyde and glycine to L-phenylalanine was conducted with resting cells of *E. coli* M8 (the OD_600nm_ of the condensed cell was 50) in 2 ml Tris buffer (100 mM, pH 8.0), 15 mM benzaldehyde and 150 mM glycine, 150 mM M formate, and 140 mM NH_4_Cl, with or without 0.5 mM NAD^+^ at 220 rpm and 30 °C for 24 h. Biotransformation of benzyl alcohol and glycine to L-phenylalanine was conducted with resting cells of *E. coli* M8 (the OD_600nm_ of the condensed cell was 50) in 2 ml Tris buffer (100 mM, pH 8.0, 15 mM benzyl alcohol, and 150 mM glycine, and 140 mM NH_4_Cl, with or without 0.5 mM NAD^+^) at 220 rpm and 30 °C for 24 h.

### Measurement of metabolites analysis

Metabolites were analyzed using an Agilent 1260 Infinity HPLC system. The target samples were collected within 24 h. The concentrations of phenylpyruvate, benzyl alcohol, and benzaldehyde, were analyzed using an Aminex HPX 87 H column (Bio-Rad, USA) and a refractive-index detector. The mobile phase is 5 mM H_2_SO_4_ with a flow rate of 0.6 mL/min. The column temperature and detection temperature are 35 ℃ and 50 ℃, respectively. The concentration of L-phenylalanine was analyzed using an Agilent C18 column (4.6 × 100 mm, 3.5 mm) and a DAD detector. The mobile phase gradient program and automated liquid sampler program were performed as the manufacturer’s instructions (http://www.chem.agilent.com/Library/applications/ 5990-4547EN.pdf).

### Plasmids construction

Primers (Table 2) were ordered from Tsingke. PCR reactions were carried out with Phanta DNA polymerase according to the manufacturer’s instructions. The sequences of all the plasmids produced were verified by DNA sequencing. The details for all plasmids are shown in Table 1. A gene fragment encoding lac repressor LacI [51] was inserted into the EcoRI site of plasmid pZE12 and pZA24 [52] to yield plasmid pZElac with ampicillin resistance, and pZAlac with kanamycin resistance, respectively.

**Table 2:** Primers used in this study

Primers	Sequence (5′ to 3′)
ltaE_P.p_ -F	AGGAGAAAGGTACCATGACAGACAAGAGCCAACAATTCGCCAGCG
ltaE_P.p_-R	AGGTCGACATAGTTAATTTCTCCTACTAGTTCAGCCACCAATGATCGTG
A8H_B.t_-F	TGGCTGAACTAGTAGGAGAAATTAACTATGTCGACCTCACCCCACCGCCCCGCTCATC
A8H_B.t_-R	ATAGTTTTGCTCATAGTTAATTTCTCCTGCTAGCTCTAGATCACGGCCACGACATG
ltaE_E.c_-F	TTAAAGAGGAGAAAGGTACCATGATTGATTTACGCAGTGATACCG
ltaE_E.c_-R	GGTGAGGTCGACATAGTTAATTTCTCCTACTAGTTTAACGCGCCAGGAATGCACGCC
ltaE_C.c_-F	AAGAGGAGAAAGGTACCTTGATGACCCAGACCGCGCCCCGCTACG
ltaE_C.c_-R	GTGAGGTCGACATAGTTAATTTCTCCTCTAAGCCACTCGCTTCAGCGCCGCGCCCAGA
ilvA_B.a_-F	AACTAGTAGGAGAAATTAACTATGTCGACTGAACAACAGGGCACCGCCCA
ilvA_B.a_-R	GTTTTATTTGATGCCTCTAGATCAGTGCTGCAGTTTCGCGTG CGCGA
TAA_B.t_-F	GTGGCTGAACTAGTAGGAGAAATTAACTATGTCTGCTCAACCCGCCTCCGACCTCG
TAA_B.tT_-R	GTTTTATTTGATGCCTCTAGATCACCCGCGCAGGAACTCGCCGTAGCGCGCG
ridA-F	TCGTGGCCGTGAGCTAGCAGGAGAAATTAACTATGAGCAAAACTATCGCGA
ridA-R	CCTTTCGTTTTATTTGATGCCTCTAGATTAGCGACGAACAGCGATCGCTTCGATC
fdh-F	AATTCATTAAAGAGGAGAAAGGTACCATGAAGATCGTTTTAGTCTT
fdh-R	CATAGTTAATTTCTCCTACTAGTTCAGCCACCAATGATCGTGCGGATATC
phedh-F	CGATAAGAAATAAACTAGTAGGAGAAATTAACTATGAGCCTGGTGGAAAAAACCAGCA
phedh-R	GTTTTATTTGATGCCTCTAGAACTAGCTTAATTACGAATATCCCATTTCGGTTTAAC
xylB_P.p_-F	AATTCATTAAAGAGGAGAAAGGTACCACCAATCCGGAGTACCGGCTTAAG
xylB_P.p_-R	ATGCTGGTTTTAGTTAATTTCTCCTGCTAGCTTAATGGAAATCAAAGCAGCAAT
VecpZE-F	GAATTCATTAAAGAGGAGAAAGGTACCCCG
VecpZE-R	TGAGCCTTTCGTTTTATTTGATGCCTCTAGACTAG
VecpZA-F	AATTCATTAAAGAGGAGAAAGGTACCAAGCTTATGTTAAAGCGT
VecpZA-R	AGCCTTTCGTTTTATTTGATGCCGCTAGCTTAGTC

#### pZE-*P*_*LlacO1*_*-ltaE*_*P.p*_*-A8H*_*B.t*_

Genes *ltaE*_*P.p*_, and *A8H*_*B.t*_ were amplified based on *Pseudomonas putida* and *Burkholderia thailandensis* genomic DNA. Primers ltaE_P.p_-F and ltaE_P.p_-R were used to amplify gene *ltaE*_*P.p*_. Primers A8H_Bt_-F and A8H_Bt_-R were used to amplify gene *A8H*_*Bt*_. The vector fragment of pZE was amplified from plasmid pIVC3 with primers VecpZE-F and VecpZE-R. Then these two fragments and the vector fragment of pZElac were digested with *Acc*65I-*Spe*I, *Spe*I-*Xba*I, and *Acc*65I -*Xba*I, respectively. These digested genes were ligated with T4 DNA ligase respectively to form the plasmid pZE- *P*_*LlacO1*_*- ltaE*_*P.p*_*-A8H*_*B.t*_.

#### pZE-*P*_*LlacO1*_*-ltaE*_*E.c*_*-A8H*_*B.t*_

Genes *ltaE*_*E.c*_, and *A8H*_*B.t*_ were amplified based on *E. coli* and *Burkholderia thailandensis* genomic DNA. Primers ltaE_E.c_-F and ltaE_E.c_-R were used to amplify gene *ltaE*_*E.c*_. Primers A8H_B.t_-F and A8H_B.t_-R were used to amplify gene *A8H*_*B.t*_. Then these two fragments were digested with *Acc*65I-*Spe*I, *Spe*I-*Xba*I. Then these three fragments and the vector fragment of pZElac were ligated with T4 DNA ligase to form plasmid pZE- *P*_*LlacO1*_*- ltaE*_*E.c*_*-A8H*_*B.t*_.

#### pZE-*P*_*LlacO1*_*-ltaE*_*C.c*_*-A8H*_*B.t*_

Genes *ltaE*_*C.c*_, and *A8H*_*B.t*_ were amplified based on *Caulobacter crescentus* CB15 and *Burkholderia thailandensis* genomic DNA. Primers ltaE_C.c_-F and ltaE_C.c_-R were used to amplify gene *ltaE*_*C.c*_. Primers A8H_B.t_-F and A8H_B.t_-R were used to amplify gene *A8H*_*B.t*_. Then these two fragments were digested with *Acc*65I-*Spe*I, *Spe*I-*Xba*I. Then these three fragments and the vector fragment of pZElac were ligated with T4 DNA ligase to form plasmid pZE- *P*_*LlacO1*_*- ltaE*_*C.c*_*-A8H*_*B.t*_.

#### pZE-*P*_*LlacO1*_*-ltaE*_*P.p*_*-ilvA*_*B.a*_

Genes *ltaE*_*P.p*_, and *IlvA*_*B.a*_ were amplified based on *Pseudomonas putida* and *Burkholderia ambifaria* genomic DNA. Primers ltaE_P.p_-F and ltaE_P.p_-R were used to amplify gene *ltaE*_*P.p*_. Primers ilvA_B.a_-F and ilvA_B.a_-R were used to amplify gene *IlvA*_*B.a*_. Then these two fragments were digested with *Acc*65I-*Spe*I, *Spe*I-*Xba*I. Then these three fragments and the vector fragment of pZElac were ligated with T4 DNA ligase to form plasmid pZE-*P*_*LlacO1*_*- ltaE*_*P.p*_*-IlvA*_*B.a*_.

#### pZE-*P*_*LlacO1*_*-ltaE*_*P.p*_*-TAA*_*B.t*_

Genes *ltaE*_*P.p*_, and *TAA*_*B.t*_ were amplified based on *Pseudomonas putida* and *Burkholderia thailandensis* genomic DNA. Primers ltaE_P.p_-F and ltaE_P.p_-R were used to amplify gene *ltaE*_*P.p*_. Primers TAA_B.t_-F and TAA_B.t_-R were used to amplify gene *TAA*_*B.t*_. Then these two fragments were digested with *Acc*65I-*Spe*I, *Spe*I-*Xba*I. Then these three fragments and the vector fragment of pZElac were ligated with T4 DNA ligase to form plasmid pZE-*P*_*LlacO1*_*- ltaE*_*P.p*_*-TAA*_*B.t*_.

#### pZE-*P*_*LlacO1*_*-ltaE*_*P.p*_*-A8H*_*B.t*_-*ridA*

Genes *ridA, ltaE*_*P.p*_, and *A8H*_*B.t*_ were amplified based on *E. coli, Pseudomonas putida* and *Burkholderia thailandensis* genomic DNA, respectively. Primers ridA-F and ridA-R were used to amplify gene *ltaE*_*P.p*_. Primers ltaE_P.p_-F-1 and ltaE_P.p_-R-1 were used to amplify gene *ltaE*_*P.p*_. Primers A8H_B.t_-F and A8H_B.t_-R were used to amplify gene *A8H*_*B.t*_. Then these two fragments were digested with *Acc*65I-*Spe*I, *Spe*I-*Nhe*I, *Nhe*I-*Xba*I. Then these three fragments and the vector fragment of pZElac were ligated with T4 DNA ligase to form plasmid pZE-*P*_*LlacO1*_*- ltaE*_*P.p*_*-A8H*_*B.t*_-*ridA.*

#### pZA-*P*_*LlacO1*_*-fdh-pdh*

Genes *phedh, fdh* were amplified based on *Bacillus badius*, and *Candida boidinii* genomic DNA, respectively. Primers phedh-F and phedh-R were used to amplify gene *phedh*. Primers fdh-F and fdh-R were used to amplify gene *fdh*. The vector fragment of pZA was amplified from plasmid pIVC3 with primers VecpZA-F and VecpZA-R. Then these two fragments and the vector fragment of pZAlac were digested with *Acc*65I-*Spe*I, *Spe*I-*Nh*eI, and Acc65I-NheI, respectively. Then these three fragments and the vector fragment of pZAlac were ligated with T4 DNA ligase to form plasmid pZA-*P*_*LlacO1*_*-fdh-phedh.*

#### pZA-*P*_*LlacO1*_*-xylB*_*P.p*_*-pdh*

Genes *xylB*_*P.p*_, *fdh* were amplified based on *Pseudomonas putida*, and *Candida boidinii* genomic DNA, respectively. Primers xylB_P.p_-F and xylB_P.p_-R were used to amplify gene *xylB*_*P.p*_. Primers fdh-F and fdh-R were used to amplify gene *fdh*. Then these two fragments were digested with *Acc*65I-*Spe*I, and *Spe*I-*Nhe*I, respectively. Then these two fragments and the vector fragment of pZAlac were homologous recombined with Exnase to form plasmid pZA-*P*_*LlacO1*_*- xylB*_*P.p*_*-fdh.*

### Supplementary Information

**Additional file 1:** **Table S1.** Bioconversion of benzaldehyde and glycine into L-phenylalanine.

## Data Availability

All data generated or analyzed during this study are included in this published article and its additional files.
