# Correction: Systematic engineering enables efficient biosynthesis of L-phenylalanine in *E. Coli* from inexpensive aromatic precursors

**DOI:** 10.1186/s12934-024-02327-y

**Published:** 2024-02-10

**Authors:** Mengzhen Nie, Jingyu Wang, Zeyao Chen, Chenkai Cao, Kechun Zhang

**Affiliations:** 1 Zhejiang University, Hangzhou 310027 Zhejiang, China; 2 Center of Synthetic Biology and Integrated Bioengineering, School of Engineering, Westlake University, Hangzhou 310030 Zhejiang, China

**Correction to: Microbial Cell Factories (2023) 23:12**

10.1186/s12934-023-02282-0

In the Acknowledgements of this article [1] the grant number relating to National Natural Science Foundation of China was incorrectly given as 10327A012001 and should have been 22078267. The original article has been corrected.
