# Supplementary material for: Systematic engineering enables efficient biosynthesis of L-phenylalanine in E. coli from inexpensive aromatic precursors
Source: Microb Cell Fact. 2024 Jan 5;23:12. doi: 10.1186/s12934-023-02282-0 (PMC10768146; doi:10.1186/s12934-023-02282-0)
Supplement: Supplementary file 1 — Additional file 1: Table S1. Bioconversion of benzaldehyde and glycine into L-phenylalanine. [file 12934_2023_2282_MOESM1_ESM.docx]

**Additional file 1**

**S****ystematic engineering enables efficient biosynthesis of L-phenylalanine in *E. coli* from inexpensive** **aromatic precursors**

Mengzhen Nie^1, 2^, Jingyu Wang^2^, Zeyao Chen^1, 2^, Chenkai Cao^1, 2^, Kechun Zhang^2^ *

*Corresponding author:

Kechun Zhang

[zhangkechun@westlake.edu.cn](mailto:zhangkechun@westlake.edu.cn)

^1^ Zhejiang University, Hangzhou, Zhejiang 310027, China

^2^ Center of Synthetic Biology and Integrated Bioengineering, School of Engineering, Westlake University, Hangzhou, Zhejiang 310030, China

**Table S1** Bioconversion of benzaldehyde and glycine into L-phenylalanine.

| Product | Enzymes | Titer  (g/L) | Conv (%) | Temperature (℃) | Time  (h) | Cofactor | Cosolvent | Source |
| --- | --- | --- | --- | --- | --- | --- | --- | --- |
| L-phenylalanine | PaTA, CgTD^F114A,R229T^  ʟ-AADH, FDH | 1.5 | 90 | 25 | 36 | 100 mM NAD^+^ | DMSO (10%) | [1] |
|  | LtaE_P.p_, A8H_B.t_  PheDH, FDH | 1.7 | 69 | 30 | 24 | 50 mM NAD^+^ | / | This work |
|  | LtaE_P.p_,CgTD^F114A,R229T^  PheDH, FDH | 1.5 | 61 | 30 | 24 | 50 mM NAD^+^ | / | This work |

**Reference**

1. Song W, Wang JH, Wu J, Liu J, Chen XL, Liu LM: **Asymmetric assembly of high-value alpha-functionalized organic acids using a biocatalytic chiral-group-resetting process.** *Nat Commun* 2018, **9:**3818.
